# Key immune cells in the tumor immune microenvironment of colorectal cancer: Roles and research advances (Review)

**DOI:** 10.3892/or.2026.9057

**Published:** 2026-01-21

**Authors:** Ming Qiu, Chongyuan Lan, Minglin Lin, Hui Ma

**Affiliations:** 1 Department of Colorectal and Anal Surgery, The First Affiliated Hospital of Guangxi Medical University, Nanning, Guangxi Zhuang Autonomous Region 530021, P.R. China; 2 Guangxi Key Laboratory of Enhanced Recovery After Surgery for Gastrointestinal Cancer, Nanning, Guangxi Zhuang Autonomous Region 530021, P.R. China; 3 Department of Research, The First Affiliated Hospital of Guangxi Medical University, Nanning, Guangxi Zhuang Autonomous Region 530021, P.R. China

**Keywords:** colorectal cancer, tumor immune microenvironment, immune cells

## Abstract

Colorectal cancer (CRC) is the third most common cancer globally and the second leading cause of cancer-related mortalities. Surgery-centered multimodal therapy remains the cornerstone of care, yet outcomes are poor in advanced or drug-resistant disease. The tumor immune microenvironment (TIME), a network of immune cells, cytokines and stromal elements, shapes antitumor immunity and can either restrain or encourage tumor growth. Specific immune cells within the TIME influence CRC biology, while immune-checkpoint blockade has delivered notable benefits, especially in microsatellite instability-high tumors. The present review discusses the principal immune cell populations in the CRC TIME, outlines their mechanisms of action and discusses emerging cell-based immunotherapies that may guide future precision treatment.

## 1. Introduction

Colorectal cancer (CRC) is one of the most prevalent malignancies of the digestive system. According to global cancer statistics, >1.9 million new CRC cases and ~900,000 mortalities due to CRC were recorded in 2022, making it the third most commonly diagnosed cancer and the second leading cause of cancer-related mortality worldwide (1). The incidence and progression of CRC are influenced by various factors, including age, genetic predisposition, lifestyle choices and environmental exposures. Because early symptoms are subtle, several patients present with advanced disease or distant spread, leading to a poor outcome (2). Currently, the mainstay of CRC treatment is comprehensive therapy centered around surgical resection, complemented by radiotherapy, chemotherapy, targeted therapy and immunotherapy. Preoperative neoadjuvant therapies and postoperative adjuvant chemotherapy or radiotherapy have improved the chances of curative outcomes. Standard adjuvant chemotherapy regimens include oxaliplatin based xeloda and oxaliplatin and folinic acid and fluorouracil and oxaliplatin regimens (3,4). Additionally, targeted therapies focusing on EGFR and vascular endothelial growth factor (VEGF) pathways have demonstrated clinical benefit in select patient populations (5,6). Nevertheless, response rates are modest and adverse effects substantial (7–9).

Immunotherapy has transformed outcomes in several malignancies and is now a key focus in CRC. Immune checkpoint inhibitors (ICIs), in particular, have shown favorable efficacy in patients with microsatellite instability-high (MSI-H) CRC. Nonetheless, ~85% of CRC cases exhibit microsatellite stability (MSS) or proficient mismatch repair (pMMR) status, and these patients typically exhibit limited responses to ICIs (10). Thus, elucidating the immune microenvironment of CRC and developing novel immunotherapeutic strategies have emerged as key areas of current research.

**Figure 1.**
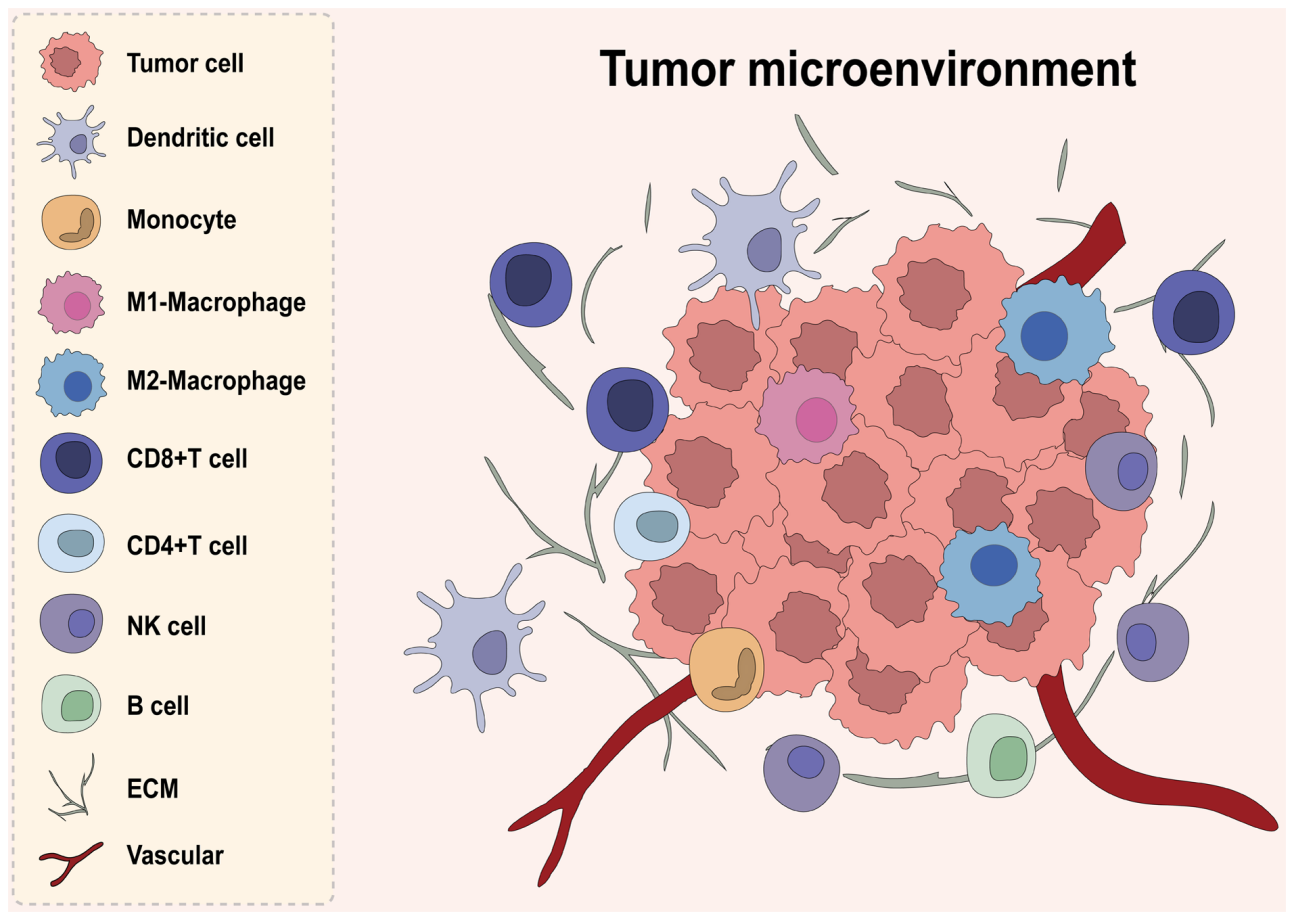
Components of the Tumor microenvironment. The cellular components within the tumor microenvironment are mainly composed of tumor cells, dendritic cells, monocytes, macrophages, T cells, NK cells, B cells and other cell types, while the extracellular matrix and cytokines constitute the extracellular components of the tumor microenvironment. NK, natural killer cell; ECM, extracellular matrix.

The tumor microenvironment (TME) comprises various components associated with tumor tissues, carrying out key roles in tumor initiation and progression. Multiple immune cells, including but not limited to macrophages, T cells, B cells, natural killer (NK) cells, dendritic cells (DCs) and neutrophils, are recruited into the vicinity of tumor cells, interacting with extracellular matrix (ECM) components and cytokines to collectively form the tumor immune microenvironment (TIME) (11) (Fig. 1). These cells can mount potent antitumor responses yet also enable immune escape. Their relative abundance and functional state help determine tumor immunogenicity, therapy response and patient survival. Intensive research has explored the roles and regulatory mechanisms of various immune cells within the CRC TIME.

## 2. Tumor-associated macrophages (TAMs)

Macrophages are abundant and versatile residents of the CRC TIME (12). TAMs interact directly with tumor cells, secreting various growth factors, cytokines and ECM-modifying enzymes, thus promoting tumor growth, migration and invasion (13). TAMs also regulate immune responses within the TME, creating an immunosuppressive milieu conducive to tumor progression by inhibiting cytotoxic T lymphocytes, expanding regulatory T cells (Tregs) and NK cell functions. Moreover, macrophages release MMPs and other ECM remodeling factors, enhancing tumor cell migration (14).

**Figure 2.**
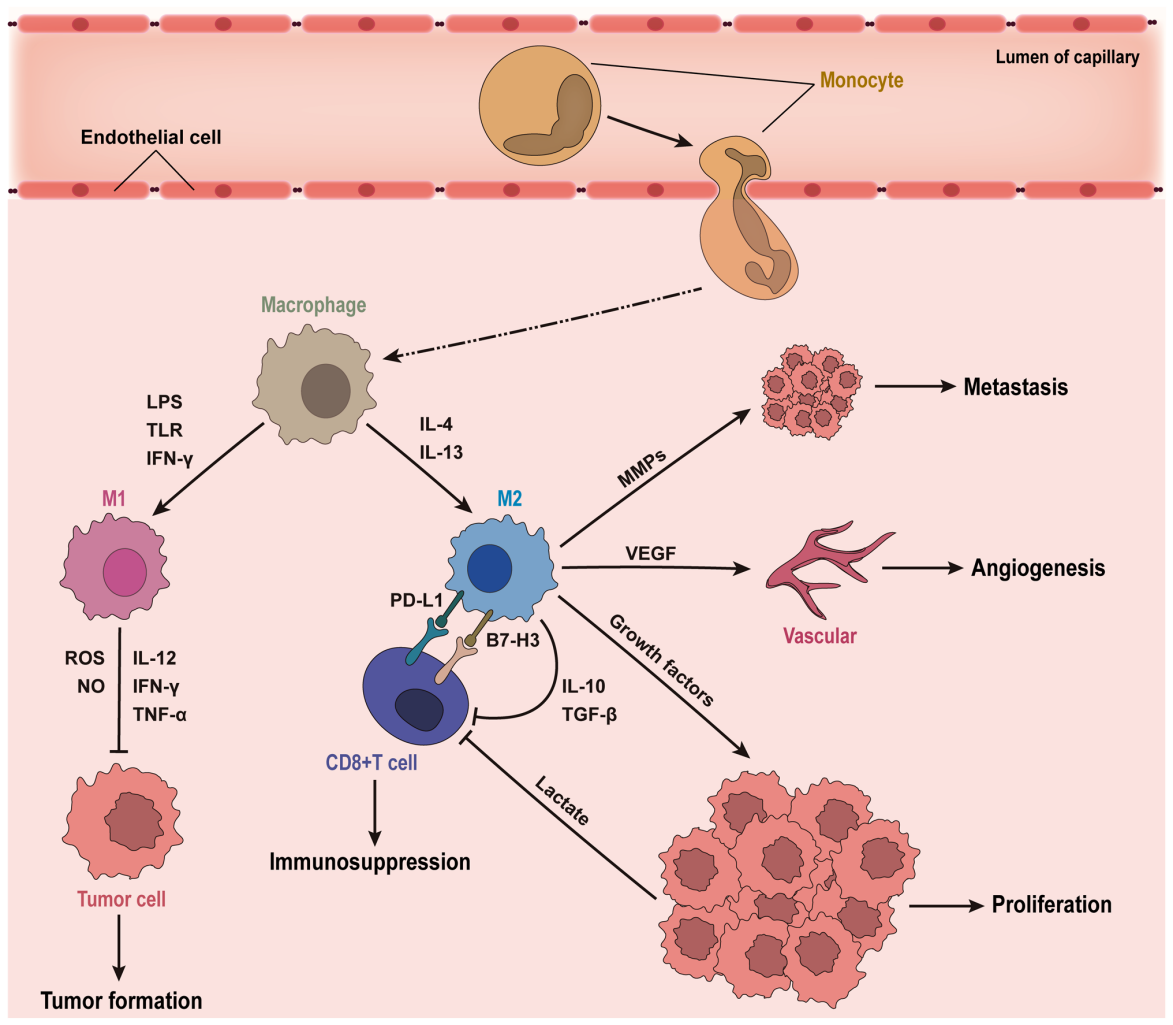
Schematic illustration of the roles of TAMs in tumor progression. After monocytes in the bloodstream migrate into the tumor tissue and differentiate into macrophages, they further polarize into M1 and M2 types of TAMs according to their distinct functional characteristics. M1-type TAMs exert cytotoxic effects against tumor cells, whereas M2-type TAMs display immunosuppressive functions and promote tumor cell proliferation, angiogenesis and metastasis formation. LPS, lipopolysaccharide; TLR, toll-like receptors; ROS, reactive oxygen species; NO, nitric oxide; VEGF, vascular endothelial growth factor.

Based on functional characteristics, TAMs are typically classified into pro-inflammatory M1 and anti-inflammatory M2 phenotypes. M1 macrophages, activated by IFN-γ, lipopolysaccharide or Toll-like receptor ligands, express markers such as major histocompatibility complex (MHC)-II, CD68, CD80 and CD86. They enhance anti-tumor immune responses through secretion of cytokines including IL-12, TNF-α and IFN-γ, and directly exert cytotoxic effects on tumor cells via reactive oxygen species and nitric oxide (NO) production (15–19). Conversely, M2 macrophages exhibit immunosuppressive roles, expressing inhibitory receptors such as programmed death-ligand 1 (PD-L1) and B7-H3 (20–22). Activated by Th2 cytokines IL-4 and IL-13, M2 macrophages express CD163, CD206 and ARG1, and secrete cytokines such as IL-10, TGF-β and VEGF, suppressing T cell activity, promoting angiogenesis and facilitating immune escape (Fig. 2) (23–25).

High infiltration of M1 macrophages associates with improved prognosis in CRC. Studies have shown an inverse relationship between the proportion of M1 macrophages and lymph node and distant metastases (26–28). Wang *et al* (29) reported that ANKRD22 expression in M1 macrophages positively associates with survival and macrophage infiltration levels in patients with colon cancer. Nussbaum *et al* (30)Q﻿ analyzing single-cell RNA sequencing (scRNA-seq) data from 23 patients with CRC, identified high SPP1-CD44 interactions within inflammatory macrophages. Targeting SPP1+ macrophages exhibiting an anti-inflammatory phenotype could disrupt these interactions, potentially reducing tumor progression and immunosuppression.

Conversely, M2 macrophage infiltration in CRC is associated with tumor progression and immune evasion. Their presence associates notably with increased tumor aggressiveness, metastatic potential and immunosuppressive capabilities (31,32). Tumor-derived exosomes serve as key mediators of intercellular communication, facilitating tumor-macrophage interactions. After internalization by macrophages, these exosomes activate polarization pathways, driving M2 macrophage polarization, creating pre-metastatic niches and subsequently promoting CRC metastasis (33–36). M2 macrophages further enhance tumor invasion and metastasis by secreting pro-tumor factors such as VEGF, MMPs, IL-4 and IL-13, contributing to angiogenesis and ECM degradation (37–39). Additionally, M2 macrophages facilitate immune escape through expression of PD-L1, engaging with PD-1 and cytotoxic T-lymphocyte associated protein 4 (CTLA-4), impairing T-cell receptor (TCR) signaling and inhibiting cytotoxic T cell function (40). They also expand Tregs via TGF-β secretion, further suppressing CD8+ T cell activities and promoting immune evasion (41).

Studies have demonstrated the dual influence of TAMs on the effectiveness of radiotherapy and chemotherapy, either antagonizing therapeutic efficacy by facilitating tissue repair and immunosuppression or enhancing overall antitumor effects (42,43). De Palma *et al* (44) found that TAMs also drive repair mechanisms following vascular-targeted therapy. Furthermore, low-dose γ irradiation of macrophages during neoadjuvant therapy can induce vascular normalization and counteract immunosuppression, diminishing pro-tumor effects of TAMs (45). Studies indicate that ICIs, such as PD-1/PD-L1 inhibitors and agents such as selicrelumab, can enhance macrophage phagocytosis, reducing tumor burden (46,47). Current clinical trials targeting TAMs primarily follow three major strategies, with differential therapeutic efficacy revealing distinct underlying mechanisms of action. First, strategy targeting the C-C motif chemokine receptor 2/C-C motif chemokine ligand 2 or colony-stimulating factor 1 receptor (CSF1R) axis represent a common therapeutic approach. This strategy inhibits TAM recruitment, effectively reducing their overall density within the TIME. Published results from clinical studies largely support the feasibility of this approach (48–51).

However, likely due to limited anti-tumor clinical activity, the majority of ongoing trials in solid tumors are Phase I/II studies focusing on safety and preliminary efficacy (50,52,53). This constrained efficacy may be partly attributed to the concurrent suppression of potentially anti-tumor M1-type TAM recruitment, thereby weakening anti-tumor immunity. Furthermore, the high plasticity of TAMs may trigger compensatory activation of other pro-tumor signaling pathways upon CSF1R inhibition, potentially restoring the pro-tumor functions of TAMs and diminishing therapeutic effectiveness (54). Second, blocking the ‘don't eat me’ signal using anti-CD47/SIRPα antibodies to activate macrophage phagocytosis constitutes another common strategy. This approach bypasses the dependency on TAM quantity and directly enhances their intrinsic tumor-killing capacity (55). Results from the NCT02953782 trial demonstrated that combined anti-CD47 and anti-EGFR therapy was tolerable and showed potential anti-tumor activity in heavily pre-treated patients with CRC, despite no notable changes in immune cell infiltration levels being observed (56). Additionally, since CD47 is involved in maintaining erythrocyte homeostasis, off-target toxicity of CD47 antibodies often limits the efficacy of monotherapy (57). Third, Chimeric Antigen Receptor Macrophage (CAR-M) therapy, which utilizes genetically engineered macrophages, represents a precision empowerment strategy. This approach not only enables specific tumor antigen recognition but may also promote macrophage polarization towards the M1 phenotype upon activation. The subsequent secretion of pro-inflammatory cytokines such as IL-12 could potentially convert ‘cold’ tumors into ‘hot’ tumors. Clinical trial results (NCT04660929) indicated that CAR-M therapy is safe and feasible in patients with HER2-overexpressing solid tumors and can induce TME remodeling and anti-tumor immune responses (58), offering a novel strategy to address prevalent immunosuppression in CRC (Table I).

Substantial advancements have been realized in the field of cancer immunotherapy over recent years (59–61). Although numerous early-phase clinical trials have explored targeting TAMs, these studies (62–64) have revealed several key and unresolved challenges: i) The lack of predictive biomarkers to prospectively identify patients who are likely to benefit; ii) difficulties in managing toxicities associated with combination therapies, as well as uncertainties regarding the optimal sequencing of treatments and their underlying mechanistic basis; iii) obstacles in genetically engineering macrophages, including their poor expansion *in vitro* and limited self-renewal capacity following adoptive transfer. Therefore, future efforts should focus on integrating specific biomarkers to identify patients most likely to benefit, and exploring combination regimens with chemotherapy, radiotherapy, immunotherapy or therapies targeting other immune cells. This strategy is essential to overcome treatment resistance and maximize therapeutic efficacy.

## 3. T cells

T cells are prominent immune cell populations within the CRC TIME, playing essential roles in tumor defense. They are categorized primarily as CD8^+^ cytotoxic T cells and CD4^+^ helper T cells based on functional differentiation. CD8^+^ T cells serve as key effectors in adaptive immunity, recognizing tumor antigens presented via MHC class I molecules and directly killing tumor cells through secretion of perforin and granzyme B (65). They also release IFN-γ and TNF-α, strengthening local immune responses and initiating apoptosis via the Fas/FasL signaling pathway (66). Increased infiltration of activated CD8^+^ T cells is associated with improved patient prognosis (67).

Multiple factors suppress CD8^+^ T cell functionality in CRC. High expression of checkpoint molecules such as PD-1 and CTLA-4 within the tumor environment induces CD8^+^ T cell exhaustion (68). In addition, the peripheral blood count of CD8^+^ T lymphocytes in patients with CRC is markedly reduced compared with that observed in healthy individuals (69). Exhausted T cells (Tex) arise from chronic antigen stimulation, exhibiting impaired responsiveness (70,71). Early-stage Tex cells retain proliferative and differentiation capacities and can partially recover with ICIs, while terminal Tex cells remain largely irreversible (72,73). Tex cells express high levels of inhibitory receptors, losing proliferative and cytotoxic abilities (74–76). Single-cell studies indicate that baseline Tex proportions and dynamic changes following PD-1 blockade can predict immunotherapy outcomes (77–79). Additional immunosuppressive factors, such as Tregs, MDSCs, TAM-derived cytokines such as IL-10, TGF-β and metabolic by-products lactate, further impair CD8+ T cell functions (25,80–82). PD-1/PD-L1 inhibitors demonstrate notable efficacy in MSI-H CRC by restoring CD8^+^ T cell activity, however, their efficacy remains limited in MSS CRC due to lower T-cell infiltration (83,84). Thus, combination therapies involving cancer vaccines or engineered T-cell therapies (CAR-T/TCR-T) might enhance antitumor responses.

CD4^+^ T helper cells modulate immune responses by regulating CD8^+^ T and B cell activities, differentiating into Th1, Th2, Th17 and Treg subsets. Th1 cells promote antitumor immunity through IFN-γ and IL-2 production (85–87), whereas Th2 cells facilitate tumor progression via pro-inflammatory cytokines IL-4, IL-5 and IL-13 (88,89). The role of Th17 cells remains controversial, potentially promoting or inhibiting CRC progression through cytokines such as IL-17 and IL-22 (90–94). Tregs, characterized by Foxp3 expression, suppress immune responses via cytokines such as TGF-β, IL-10 and IL-35, associating with poor prognosis and increased metastasis in CRC (95–100). Thus, targeting Treg metabolism or TGF-β pathways may enhance anti-tumor immunity in CRC.

## 4. B cells

B cells represent a key component of adaptive immunity, mediating anti-tumor responses primarily through antibody production, antigen presentation and the promotion of T cell activation (101). The antibodies secreted by B cells can bind to tumor cells, facilitating cytotoxic activities mediated by NK cells, macrophages and the complement system. Additionally, B cells function as antigen-presenting cells, stimulating CD4+ T cells via MHC class II molecules, thereby enhancing antitumor immune responses (102). However, the role of B cells within the TME is not exclusively protective.

Certain subsets of B cells, particularly regulatory B cells (Bregs), may promote tumor progression (103). Bregs secrete immunosuppressive cytokines such as IL-10 and TGF-β, inhibiting CD8^+^ T cell and NK cell functions, thus facilitating tumor immune escape (104). Moreover, B cells can exacerbate tumor-associated inflammation by producing pro-inflammatory cytokines, including IL-6, thereby enhancing cancer cell proliferation and metastasis (105). Clinical studies demonstrate that increased infiltration of Bregs in CRC tissues associates positively with tumor progression and predicts poor patient prognosis (106,107).

Targeting B cells for immunomodulation has emerged as a novel direction for CRC treatment. On one hand, enhancing antigen-specific B cell responses, such as developing B-cell-based vaccines, might potentiate antitumor immunity (108). On the other hand, inhibiting Breg functions to reduce their immunosuppressive effects could improve responses to immunotherapy. Monoclonal antibodies targeting Breg-specific markers, such as CD19 or CD38, have been proposed as promising therapeutic approaches (109,110). Additionally, interactions between B cells and T cells are key within the CRC immune microenvironment (111). Thus, future therapeutic strategies should comprehensively consider dynamic interactions among various immune cell subsets to optimize immunotherapy outcomes.

## 5. NK cells

NK cells constitute essential components of innate immunity, capable of directly recognizing and killing tumor cells independently of antigen presentation. NK cells induce apoptosis in target cells through the secretion of perforin and granzyme B, and enhance antitumor immune responses via IFN-γ production (112). Additionally, NK cells contribute markedly to antibody-dependent cellular cytotoxicity, enhancing the efficacy of antibody-based therapies (113). NK cells are principally stratified into two distinct functional subsets contingent upon the surface density of CD56 expression: The CD56dim and CD56bright subpopulations. The former constitutes the numerically predominant fraction, characterized by potent cytotoxicity and serving as the primary effector killer cells; in contradistinction, the latter subpopulation is specialized chiefly in cytokine secretion and the orchestration of immune responses (114).

NK cell activity is notably impaired. CRC tissues often exhibit reduced NK cell infiltration and functionality due to immunosuppressive factors present within the TME (115). For example, cytokines such as TGF-β and IL-10, abundant in the CRC microenvironment, directly inhibit NK cell cytotoxicity, inducing a state of functional exhaustion (116,117). Furthermore, increased expression of inhibitory receptors, such as PD-1 on NK cells within CRC tumors, reduces their capacity to recognize and eliminate tumor cells, thereby allowing tumor cells to evade immune surveillance (118). The modalities underpinning this immune escape are multifaceted. Initially, CRC neoplastic cells elude immunosurveillance mediated by the NK activating receptor NKG2D via the specific downregulation of MHC-I related molecular expression (119). Concomitantly, NK cells exhibit elevated surface expression patterns of novel immune checkpoint receptors beyond PD-1, namely NKG2A/CD94, TIGIT and TIM-3. The cognate ligands for these inhibitory receptors are abundantly expressed by tumor cells or residing within the TME, collaboratively engendering a state of profound NK exhaustion phenotype (120). Moreover, the deleterious metabolic milieu within the TME constitutes another key determinant: Overexpressed CD73 enzymatically catabolizes extracellular ATP into adenosine, which subsequently engages the A2AR receptor on the NK cell surface, thereby abrogating their proliferative capacity and cytotoxic effector functions (121).

Strategies to enhance NK cell antitumor activities include CAR-NK cell therapy, NK cell stimulants, such as IL-15 and combination treatments involving PD-1/PD-L1 blockade (122). Preclinical studies indicate CAR-NK cells exhibit potent antitumor activity and possess superior safety profiles compared with CAR-T cell therapies (123–125). However, due to challenges associated with NK cell proliferation and survival *in vitro*, further optimization of NK cell-based immunotherapies remains essential to improve their clinical utility in CRC treatment.

## 6. DCs

DCs, originating from hematopoietic stem cells in the bone marrow, are the most potent professional antigen-presenting cells. DCs capture exogenous antigens, process them into antigenic peptides and present them in complex with MHC molecules on their surface, initiating naïve T cell activation. DCs are a highly heterogeneous group, primarily categorized into conventional DCs (cDCs) and plasmacytoid DCs (pDCs). Functionally, cDCs specialize in antigen processing and presentation, while pDCs are less proficient in antigen presentation but produce substantial amounts of type I interferons (126).

Within the TME, DCs serve as pivotal antigen-presenting cells, capturing, processing and presenting tumor antigens to activate T cell-mediated antitumor immune responses. DCs stimulate CD4^+^ T cells via MHC-II molecules and also facilitate CD8^+^ T cell activation through cross-presentation (127). cDCs constitute the predominant DC population within the TME and are further delineated into distinct cDC1 and cDC2 subsets. Notably, the cDC1 subpopulation, whose development is dependent upon the transcription factor BATF3, assumes an indispensable role in orchestrating anti-tumor T cell immune responses (128), whereas cDC2s are principally tasked with initiating Th2-type immunity. However, DC functionality in the CRC microenvironment is frequently compromised, impairing effective antitumor immunity. Immunosuppressive factors, such as prostaglandin E2 and IL-6, inhibit DC maturation, thereby limiting their capacity to activate T cells effectively. This specific functional deficit is particularly salient within the context of CRC. Investigations have elucidated that in MSS CRC phenotypes, aberrant intrinsic activation of the Wnt/β-catenin signaling axis constitutes a pivotal mechanism underpinning immunosuppression (129). Mechanistically, this pathway acts to repress the expression of chemokines CCL4 and CCL5, thereby specifically impeding the efficacious intratumoral infiltration of the key cDC1 subset; this cascade consequently renders the TME an immunologically ‘cold’ landscape, ultimately conferring primary resistance to ICIs (130). Additionally, some DC populations may acquire tolerogenic properties, failing to effectively present antigens and instead promoting Treg expansion, further suppressing antitumor responses (131).

Current strategies aiming to restore DC-mediated antitumor immunity include DC vaccines, autologous DC infusion and combination therapies with ICIs (132,133). DC vaccines hold promise for CRC treatment, with several clinical trials currently evaluating their efficacy in combination with ICIs. Furthermore, prospective therapeutic avenues could involve targeted intervention against the Wnt/β-catenin signaling axis to ameliorate the established impediments to cDC1 recruitment within the TME, thereby orchestrating the conversion of immunologically ‘cold’ tumors into a ‘hot’ phenotype (134–136). This strategy could subsequently be leveraged in synergistic combinations with ICIs to augment overall antitumor therapeutic efficacy.

## 7. Neutrophils

Neutrophils represent a primary barrier of innate immunity, carrying out key roles in inflammation and immune responses. Within the TME, neutrophils can be further categorized into N1 phenotypes, which exert anti-tumor effects and N2 phenotypes, which facilitate tumor progression. Growth factors, cytokines and chemokines present in the TME recruit neutrophils into the tumor tissue, where they participate in the regulation of diverse and complex biological processes (137). Prevailingly, elevated concentrations of TGF-β function as a cardinal impetus driving the phenotypic skewing of neutrophils from an N1 state towards the pro-tumorigenic N2 phenotype (138). N2-polarized neutrophils predominantly manifest immunosuppressive functionalities, exerting these effects via the regulated secretion of a complex array of chemokines, pro-angiogenic factors and immunosuppressive moieties. Consequently, this bioactive secretome actively recruits and activates collateral immunosuppressive cell populations, facilitates neovascularization and concurrently subverts host immune system recognition and cytotoxic clearance of tumors (139).

Neutrophils carry out a key role in the metastatic process of CRC. Under specific signaling stimuli, neutrophils undergo a unique form of cell death known as neutrophil extracellular trap (NET) formation. The released NETs not only physically capture circulating tumor cells (140), but studies have also revealed that NETs interact via histones on DNA with specific receptors on tumor cell surfaces, activating downstream signaling pathways that promote the colonization and survival of tumor cells in distant organs (141,142). Furthermore, NETs facilitate immune evasion by degrading anti-tumor molecules within the TME (143). Clinical evidence further confirms that an elevated peripheral blood neutrophil-to-lymphocyte ratio serves as an independent predictor of poor prognosis and resistance to immunotherapy in patients with CRC (144).

Therapeutic approaches targeting neutrophils include inhibition of NET formation and blockade of the CXCR2 signaling pathway (145,146), with the objective of diminishing neutrophil recruitment within the TME and augmenting the efficacy of immunotherapeutic interventions. Consequently, the strategic modulation of neutrophil polarization states, the suppression of NET generation and the targeted disruption of their recruitment signals represent viable avenues for enhancing therapeutic outcomes in CRC management.

## 8. Application of multi-omics technologies in CRC TIME

Multi-omics approaches integrate multiple molecular analyses to elucidate interactions among various biological components. Recently, notable advancements in scRNA-seq and spatial transcriptomics have profoundly enhanced the understanding of the CRC TIME (77,147,148), illuminating immune cell heterogeneity, functional states and interactions with tumor cells, thereby offering novel insights into CRC pathophysiology.

ScRNA-seq provides comprehensive information at the transcriptomic, genomic and epigenomic levels for individual cells, enabling researchers to identify distinct immune cell subsets within the TME, characterize their functional states and reveal potential intercellular communication pathways. Zhang *et al* (149) conducted a detailed scRNA-seq analysis of tumor-infiltrating myeloid cells in patients with CRC, elucidating the characteristics and lineage trajectories of TAM and DC subpopulations, as well as their interactions with T cells and other cell types. Their study revealed a specific enrichment of secreted phosphoprotein 1+ (SPP1+) TAMs in CRC tissues and demonstrated the similarity between CSF1R inhibitor-resistant TAM subsets and SPP1+ TAM populations, providing a mechanistic explanation for the limited efficacy of anti-CSF1R monotherapy and highlighting the role of SPP1+ macrophages in promoting tumor metastasis (149,150). Importantly, the identification of this SPP1+ TAM subset provides the molecular evidence for the therapeutic resistance mentioned in the TAM section, validating that the compensatory activation limiting CSF1R inhibitor efficacy is driven by distinct, intrinsically resistant macrophage subpopulations. The investigation by Zhang *et al* (149) had elucidated, at single-cell resolution, a landscape of TAM heterogeneity that was markedly more intricate when compared with the canonical M1/M2 stratification paradigm. This had substantially augmented the comprehension of intratumoral TAM diversity by precisely pinpointing an SPP1+ TAM subset exhibiting inherent resistance to CSF1R inhibitors. This key discovery not only offers a mechanistic rationale for the aforementioned therapeutic predicaments but further delineated that prospective targeting avenues must focus on the precise therapeutic interrogation of such distinct pro-tumorigenic subpopulations. Additionally, Wu *et al* (151) applied combined scRNA-seq and spatial transcriptomics to CRC liver metastasis samples, systematically illustrating spatial expression patterns of SPP1+ macrophages and mannose receptor C-type 1+, CCL18+ M2-like macrophages, suggesting a key immunosuppressive role of the TAM in CRC liver metastasis (151).

Moreover, Zhang *et al* (152) performed comprehensive analyses of CRC-associated T cells via single-cell sequencing, thoroughly characterizing their tissue distribution, clonality, migration and dynamic transitions. Through integrated single-cell TCR sequencing, they discovered that both the TME and TCR repertoire influenced the differentiation of tumor-infiltrating CD8+ effector-memory T cells toward Tex and Treg. This high-resolution trajectory analysis refines the concept of the aforementioned T-cell exhaustion, demonstrating that the shift from effector to terminal exhausted states is a dynamic continuum governed by specific TCR repertoires rather than a static classification. This finding had profoundly augmented the comprehension regarding the dynamics of T cell dysfunction, thereby furnishing a theoretical underpinning for their inherent plasticity and potential for reversibility. Further studies identified that Tex or tumor-reactive-like CD8+ T (Ttr-like) cells were associated with the efficacy of anti-PD-1 immunotherapy (78,79). Specifically, a higher abundance of blood-associated Ttr-like cells exhibiting exhaustion phenotypes associated with improved therapeutic outcomes (77). Another notable study integrating spatially enhanced resolution omics sequencing and scRNA-seq identified physical interactions and abundance of lysosomal associated membrane protein 3+ DCs and CXCL13+ T cells, potentially shaping an immune checkpoint blockade (ICB) responsive tumor-stroma boundary. This spatial arrangement and immune status were distinctly characterized dMMR and pMMR CRC and were considerably associated with responses to ICB therapy (153). Collectively, these observations underscore that the therapeutic efficacy of ICB was not predicated merely upon the quantitative abundance of immune infiltrates, but rather hinges upon the spatially orchestrated crosstalk between distinct functional T cell subsets and specific antigen-presenting cells. This spatial organization constituted the structural architectural basis governing ICB outcomes. Such findings proffered a profound mechanistic rationale elucidating why treatment efficacy from immunotherapy remain restricted to a select fraction of the patient population.

The advancement and integrated application of multi-omics technologies have shown considerable potential in dissecting immune cell composition, functional dynamics and tumor-immune cell interactions within the CRC microenvironment. Such comprehensive insights provide key foundations for developing novel therapeutic strategies and advancing personalized medicine.

## 9. Limitations and challenges in translating TIME-targeted therapies to CRC

Notwithstanding the remarkable preclinical strides in TIME-targeted immunotherapies, translating these findings into tangible clinical benefits for patients with CRC is thwarted by multifaceted impediments, principally stemming from the profound heterogeneity of the CRC microenvironment (77). While ICIs demonstrate efficacy in MSI-H subsets, the vast majority of patients with MSS exhibit an ‘immune desert’ or ‘immune exclusion’ phenotype. This intrinsic immunological dormancy renders single-agent TIME-directed strategies insufficient to revert their immune-silent status. Moreover, the dynamic adaptability of the TIME is another key determinant of therapeutic failure. The TME possesses a high degree of compensatory capacity. This mechanism of compensatory immune evasion severely curtails the durable efficacy achievable with monotherapies.

The safety profile and the predictive value of preclinical models also urgently warrant resolution. Several TIME targets, such as CD47 and TGF-β, are ubiquitously expressed in healthy tissues, meaning that off-target systemic toxicity severely constrains the therapeutic window for these agents (154,155). While combinatorial regimens are deemed the principal strategy to circumvent resistance, this approach invariably precipitates compounding toxicities and an escalated risk of immune-related adverse events. Concomitantly, clear clinical guidance remains lacking for the precise determination of the optimal sequencing for distinct therapeutic agents. Furthermore, the widely employed subcutaneous xenograft models exhibit rapid growth dynamics and lack the complex human CRC stromal architecture and the long-term co-evolutionary interplay between the immune system and the tumor (156). The disparity between these models and clinical pathological features often results in a failure to recapitulate efficacy in human trials, despite promising results observed in mice. Therefore, future research should focus on developing more clinically relevant models and exploring combination therapy strategies to overcome the limitations of targeting single pathways.

## 10. Discussion

Accumulating evidence indicates that tumor progression involves not only the intrinsic characteristics of tumor cells but also key interactions within the TIME (157,158). Immune cells within the TIME carry out pivotal roles in CRC initiation, progression and immune evasion processes. Specifically, these immune cells shape the host immune responses, considerably influencing tumor development, thereby positioning the TIME as a promising therapeutic target for cancer treatment. Immunotherapy has evolved from simply enhancing immune effector functions to specifically targeting immunosuppressive factors within the TIME. Consequently, strategies aimed at effectively modulating the TIME to amplify the therapeutic effects of immunotherapy are emerging as a key area of ongoing and future research.

To advance the transition from targeting single cells toward systematically reshaping the TIME through combination therapies, future research should focus on multidisciplinary integration and overcoming challenges such as drug resistance and the limitations of immunotherapy, thereby enabling the development of personalized treatment regimens. Although patients with dMMR/MSI-H CRC benefit from ICIs, the majority of CRC cases are pMMR/MSS and remain unresponsive to ICI-based immunotherapy (10). Therefore, a primary objective is to overcome the immunosuppressive state in pMMR/MSS CRC by enhancing immunogenicity to render these tumors susceptible to immunotherapy. For instance, immunosuppressive M2-type TAMs express inhibitory ligands such as PD-L1 and B7H3, which contribute to immune suppression and diminish the efficacy of T cell-directed ICIs. Combining therapies that target these immunosuppressive TAMs with PD-1/PD-L1 blockade is thus a rational strategy. Clinical evidence supports this approach: The combination of the CSF1R inhibitor emactuzumab with the anti-PD-L1 agent atezolizumab has demonstrated a notable objective response rate in ICI-naïve patients (159). This provides a promising avenue for improving outcomes in pMMR/MSS patients with CRC. Furthermore, breakthroughs in drug development technologies and insights from spatial transcriptomics into TIME-mediated immunosuppression and resistance mechanisms will be instrumental in achieving precision medicine and addressing treatment insensitivity in a subset of patients with CRC. For example, a previous study showed that deep learning can predict MSI status directly from routine histology slides with high concordance with standard molecular testing (160), underscoring the potential of multidisciplinary integration to inform individualized precision therapy.

Comprehensive understanding of the composition and functional dynamics of the TIME, along with its interplay in CRC development and immune escape mechanisms, can provide key insights into the underlying pathogenesis of CRC. Such knowledge offers a robust theoretical framework for developing novel immunotherapeutic approaches. Future research dedicated to unraveling the regulatory mechanisms and interactive networks within the TIME will facilitate individualized, precision-based therapeutic strategies, ultimately improving both survival outcomes and quality of life for patients with CRC.

## Figures and Tables

**Table I.** Clinical trials of macrophage-based drugs in solid tumors or colorectal cancer.

NCT number	Agent name	Target	Drug type	Phase	Conditions
NCT01316822	ARRY-382	cFMS	cFMS inhibitor	1	Metastatic cancer
NCT04660929	CT-0508	Her-2	CAR macrophages	1	HER2 overexpressing solid tumors
NCT01204996	CNTO 888	CCL2	Anti-CCL2 monoclonal antibody	1	Solid tumors
NCT02777710	Pexidartinib	CSF1R	Anti-CSF1R	1	Pancreatic or colorectal cancer
NCT02880371	ARRY-382	CSF1R	CSF1R inhibitor	1, 2	Advanced solid tumors
NCT01346358	IMC-CS4	CSF1R	Anti-CSF1R	1	Advanced solid tumors
NCT02718911	LY3022855	CSF1R	CSF1R inhibitor	1	Advanced solid tumors
NCT03158272	Cabiralizumab	CSF1R	CSF1R antagonist	1	Advanced malignancies
NCT02526017	Cabiralizumab	CSF1R	CSF1R antagonist	1	Advanced solid tumors
NCT02323191	Emactuzumab	CSF1R	CSF1R antagonist	1	Advanced solid tumors
NCT02760797	Emactuzumab	CSF1R	CSF1R antagonist	1	Advanced solid tumors
NCT01494688	Emactuzumab	CSF1R	CSF1R antagonist	1	Advanced solid tumors
NCT03993873	Elzovantinib	CSF1R	CSF1R antagonist	1	Advanced solid tumors
NCT03069469	Vimseltinib	CSF1R	CSF1R antagonist	1, 2	Advanced solid tumors
NCT04648254	Q702	CSF1R	CSF1R antagonist	1	Advanced solid tumors
NCT03238027	Axatilimab	CSF1R	CSF1R antagonist	1	Solid tumors
NCT03783403	CC-95251	SIRPα	Anti-SIRPa monoclonal antibody	1	Advanced solid tumors
NCT03990233	BI 765063	SIRPα	Anti-SIRPa monoclonal antibody	1	Advanced solid tumors
NCT02216409	Magrolimab	CD47	Anti-cd47 monoclonal antibody	1	Solid tumors
NCT02953782	Magrolimab	CD47	Anti-cd47 monoclonal antibody	1, 2	Advanced colorectal cancer

CSF1F, colony-stimulating factor 1 receptor; SIRPα, signal regulatory protein α; cFMS, cellular feline mcdonough sarcoma.

## Data Availability

Not applicable.
